# Transcriptome Analyses from Mutant *Salvia miltiorrhiza* Reveals Important Roles for *SmGASA4* during Plant Development

**DOI:** 10.3390/ijms19072088

**Published:** 2018-07-18

**Authors:** Hongbin Wang, Tao Wei, Xia Wang, Lipeng Zhang, Meiling Yang, Li Chen, Wenqin Song, Chunguo Wang, Chengbin Chen

**Affiliations:** College of Life Sciences, Nankai University, Tianjin 300071, China; 2120150901@mail.nankai.edu.cn (H.W.); 8190537@nankai.edu.cn (T.W.); 2120160946@mail.nankai.edu.cn (X.W.); 1120140344@mail.nankai.edu.cn (L.Z.); meilingyang@tju.edu.cn (M.Y.); lichen@nankai.edu.cn (L.C.); songwq@nankai.edu.cn (W.S.)

**Keywords:** transcriptome, *Salvia militiorrhiza*, mutant, *Arobidopsis*, *GASA*, GA, drought, salt, paclobutrazol

## Abstract

*Salvia miltiorrhiza* (*S. miltiorrhiza*) is an important Chinese herb that is derived from the perennial plant of Lamiaceae, which has been used to treat neurasthenic insomnia and cardiovascular disease. We produced a mutant *S. miltiorrhiza* (MT), from breeding experiments, that possessed a large taproot, reduced lateral roots, and defective flowering. We performed transcriptome profiling of wild type (WT) and MT *S. miltiorrhiza* using second-generation Illumina sequencing to identify differentially expressed genes (DEGs) that could account for these phenotypical differences. Of the DEGs identified, we investigated the role of *SmGASA4*, the expression of which was down-regulated in MT plants. *SmGASA4* was introduced into *Arobidopsis* and *S. militiorrhiza* under the control of a CaMV35S promoter to verify its influence on abiotic stress and *S. miltiorrhiza* secondary metabolism biosynthesis. *SmGASA4* was found to promote flower and root development in *Arobidopsis*. *SmGASA4* was also found to be positively regulated by Gibberellin (GA) and significantly enhanced plant resistance to salt, drought, and paclobutrazol (PBZ) stress. *SmGASA4* also led to the up-regulation of the genes involved in salvianolic acid biosynthesis, but inhibited the expression of the genes involved in tanshinone biosynthesis. Taken together, our results reveal *SmGASA4* as a promising candidate gene to promote *S. miltiorrhiza* development.

## 1. Introduction

*S. miltiorrhiza* is considered the model Chinese medicinal plant. The demand for *S. miltiorrhiza* in both Asia and Western countries is large due to its documented therapeutic benefits. The roots and rhizomes of *S. miltiorrhiza* are used to treat neurasthenic insomnia, cardiovascular disease, cerebrovascular disease, hyperlipidemia, and acute ischemic stroke [1,2]. The active ingredients of *S. miltiorrhiza* can be divided into two groups, namely: (1) lipid-soluble tanshinones including tanshinone I, tanshinone IIA and tanshinone IIB, cryptotanshinone, dihydrotanshinone I; (2) hydrophilic phenolic acids, including rosmarinic acid, salvianolic acid B, lithospermic acid and dihydroxyphenyllactic acid, phenolic acid that displays antibacterial, anti-oxidative, and antiviral activity [3,4]. The medical component of *S. miltiorrhiza* is primarily concentrated on the surface of its roots. The biosynthetic production of these therapeutic compounds has been extensively studied in *S. miltiorrhiza*, but few reports have focused specifically on the root development [5,6]. Improving the current protocols to maximize *S. miltiorrhiza* development is therefore important. Several external factors are known to influence *S. miltiorrhiza* root development, including the NH_4_^+^:NO_3_^−^ ratio [7] and the inclusion of the fungus *Alternaria* sp. *A13*, which markedly enhances *S. miltiorrhiza* root growth [8]. However, the genetic requirements that regulate *S. miltiorrhiza* growth in vivo are less well understood. In this regard, we previously identified mutant plants (MT) produced during the breeding experiments that possessed large taproots (2.85 times of the wild type (WT)) reduced lateral roots (0.44 times lateral root number of the WT), the ratio of lateral root compare with taproot (MT 18.75%, WT 55.36%) and defective flowering, the MT with defect corolla and stamen, and can’t form normally. We reasoned that the identification of the genetic basis for this MT phenotype may reveal the genetic factors that contribute to root development which could be used to direct future *S. miltiorrhiza* breeding strategies [9,10].

Several transcriptome analyses have been performed in *S. miltiorrhiza* that were designed to identify the biosynthetic processes that regulate the production of bioactive compounds in a range of tissues, or in response to specific induction stimuli [11,12]. However, the transcriptome analysis that focuses on root development and root-specific compound production is less well understood.

GASA (Gibberellic Acid-Stimulated *Arabidopsis*) belongs to the family of cysteine-rich peptides (CRPs) [13]. GASA is widely distributed among plant species displaying organ specific tissue expression and temporal specificity. The subcellular localization of GASA varies amongst the different plant members, but it is thought to primarily distribute to the cell wall [14], and is regulated by GA [15]. *GAST1* (GA-stimulated transcript 1) was the first identified gene; *gib1* is a GA receptor loss mutant and its expression was induced by the application of exogenous gibberellin [11]. The *GASA* gene family has also been identified in *Petunia hybrid* [14], *Arabidopsis thaliana* [12,16], *Solanum tuberosum* [17,18], and *Oryza sativa* [19]. The *GASA* genes products play an important role in plant seed germination [20], lateral root formation [21], stem length [22,23], flowering [23,24], flower and fruit development [25], biotics [26,27], abiotic stress [12,28], and the ability to respond to and translate phytohormone signals [28].

The *GASA* genes contain conserved stretches of 12 cysteine residues [11,29], which are thought to be required for the generation of the structural elements that regulate protein–protein interactions [14]. In addition, up to five disulfide bridges can form across these cysteine rich regions [30], potentially regulating the redox status [30]. The loss of these cysteine stretches leads to a loss of *GASA* structural integrity and function [31,32]. The *GASA* promoter sequence includes 1–4 GARE (Gibberellin Acid Response Element) components and 1–5 ABRE (Abscisic Acid Response Element), which participate in the GA and ABA pathways, respectively. This implies that the *GASA* expression is regulated by both GA and ABA [12]. GA promotes plant growth and development, flowering, and capsule formation, and, in synergy with auxin, can regulate *Populus* root growth and development [29]. *GASA* is a downstream target of GA [33], which is regulated through the DELLA (GA Insensitive-GAI and RGA) protein dependent and independent mechanisms.

Analyses of the *GASA1*, *GASA3*, *GASA4*, *GASA8*, *GASA10*, and *GASA14* promoters reveals their activity in root tissue and the vasculature, although through distinct spatial and temporal patterns. Roles in other tissues, including meristems, flowers, and seeds, have also been reported. *AtGASA4* regulates the flower stem tissue [32], promotes lateral root formation [17,18], positively regulates seed size and products, and promotes seed germination through GA activation. The overexpression of *AtGASA4* in *Arobidopsis* reduces ROS (reactive oxygen species) accumulation and promotes GA activity during the H_2_O_2_ response [31]. The overexpression of *AtGASA14* in *Arobidopsis* also inhibits oxygen accumulation, and plants with *gasa14* mutations show sensitivity to NaCl and ABA [14]. Using yeast hybrid screens, Ceserani et al. demonstrated the interaction of *AtGASA4* and *AtGASA14* with cytoplasmic structures, implicating their involvement during plant growth [34].

Approximately 12,824 *CRP* (cysteine rich peptides) genes have been identified in 33 species, and 445 of these genes code for GASA/Snakin proteins [11]. Despite this knowledge, in vivo studies on the functional relevance of these genes is lacking. Although the *GASA* family of genes are important to plants, their roles in *S. miltiorrhiza* remain poorly characterized. In this study, to reveal the mechanism of the MT phenotype and to identify new *S. miltiorrhiza* breeding genes, we employed RNA-seq to comparatively analyze the functional complexity of the *S. miltiorrhiza* transcriptome in WT vs. MT plants. We identified a *GASA* family gene termed *SmGASA4,* from the *S. militiorrhiza* transcriptome that encodes a 110-amino acid protein, the expression of which is inhibited in MT plants. Herein, we have introduced *SmGASA4* into *Arobidopsis* and *S. militiorrhiza* under the control of a CaMV35S promoter, to verify its role during GA stimulation, drought, salt, and paclobutrazol (PBZ) stress, and as a secondary metabolism. We aimed to reveal the in vivo functions of *SmGASA4* and its potential benefits to *S. militiorrhiza* breeding.

## 2. Results

### 2.1. Transcriptome Analysis of S. militiorrhiza

#### 2.1.1. Illumina Sequencing and De Novo Assembly

To gain a comprehensive overview of the root development in *S. miltiorrhiza*, we performed a transcriptome analysis of WT and MT plants. To enhance the data stability, biological repeats of each of the samples were performed. The samples were sequenced using an Illumina HiSeq™ 2500 with the production of 100 bp paired-end reads. After stringent data filtering and quality assessment, 27,255,174 clean paired-end reads were produced from the MT, as well as 30,598,611 reads from the WT plants (Table S1). The Q30 (probability of incorrect base call <1‰) percentages and GC percentages are illustrated in Table S1, which showed Q30 percentages of 91.58% (MT) and 91.83% (WT) (Table S1). We acquired 11.69 GB of high quality clean-data from the two libraries, assessed 50,587 Unigenes through the Trinity program assemble, and achieved N50 lengths of 1540 bp (MT) and 1610 bp (WT). The mean lengths were 908.75 bp for the MT, and 901.36 bp for the WT plants (Figure S1, Table S2).

The matched sequences were analyzed using the unified Transcript or Unigenes library. We obtained 27,255,174 clean reads and 21,538,490 (79.02%) mapped reads from the MT plants, and 30,598,611 clean reads and 24,135,294 (78.87%) mapped reads from the WT plants (Table S3).

#### 2.1.2. Unigene Annotation and Functional Classification

The unigenes from the MT and WT samples were aligned to the non-redundant (nr), Swiss-Prot, gene ontology (GO), Clusters of Orthologous Groups (COG), euKaryotic Orthologous Groups (KOG), and Kyoto Encyclopedia of Genes and Genomes (KEGG) databases. We used BLAST software with parameter *E*-values less than 10^−5^ and HMMER (Biosequence analysis using profile hidden Markov Models) parameter *E*-values less than 10^−10^ to investigate the function. Once complete, the Unigene amino acid predictions were performed using HMMER software to compare the Unigene sequences with the Pfam libraries to achieve the Unigene annotated information. Among the 50,587 unigenes, 30,000 (59.3%) were annotated and 20,587 were not-documented. This may be due to technical limitations, including read length and sequencing depth, or the specificity of the *S. miltiorrhiza* genes. Amongst the annotated unigenes, 10,115 (20%), 20,532 (40.6%), 6337 (12.5%), 17,343 (34.3%), 21,674 (42.8%), 21,304 (42.1%), and 29,805 (58.9%) were annotated in the COG, GO, KEGG, KOG, Pfam, Swiss-Prot, and nr databases, respectively (Table S4).

**Figure 1:**
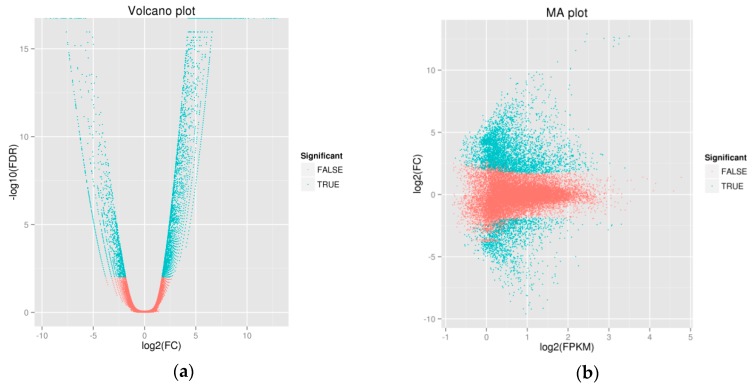
(**a**) Volcano plots of the differentially expressed genes (DEGs) in the comparisons of wild type (WT) and mutant type (MT) plants, (**b**) MA (Minus Average) plots of DEGs in the comparisons of WT and MT plants.

**Figure 2:**
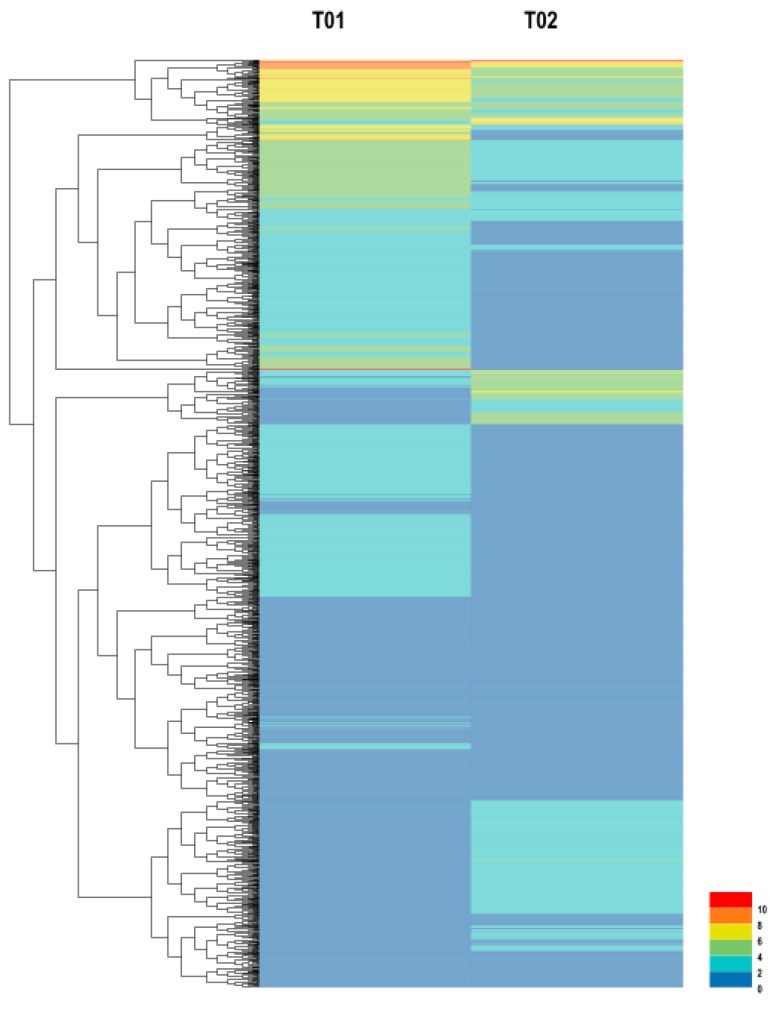
Cluster analysis of DEGs in WT and MT plants, based on expression profiles measured by RNA-seq; the color scale in the heat map corresponds to log2 (Fragments Per Kilobase Million (FPKM)) values of genes in the different samples.

We analyzed the unigene expression in the WT and MT plants using DESeq software, by normalizing the values to Fragments Per Kilobase Million (FPKM). We used the Benjiamini–Hochberg method to verify and revise the *p*-values. Using FDR values of <0.01, the fold-changes (FC) ≥ 2 were classed as differentially expressed genes. The comparison of WT and MT plants revealed 3714 up-regulated genes and 1371 down-regulated genes (Table S5). Volcano plots were created to demonstrate the fold changes in the expression and statistical comparisons (Figure 1a). MA (Minus Average) plot was used to assess the gene expression abundance and the differences in the whole distributions between the samples (Figure 1b). There were 4761 differentially expressed genes (DEGs) that were annotated in the volcano plot and MA. When comparing the WT and MT plants, 3440 up-regulated as opposed to 1321 down-regulated genes were identified. The hierarchical clustering analysis was used to screen the DEGs and cluster them according to the expression behavior under different experimental conditions (Figure 2). From the comparison of the DEG categories, 3714 were down-regulated and 1371 were up-regulated after the WT mutated to MT (Table S5).

**Figure 3:**
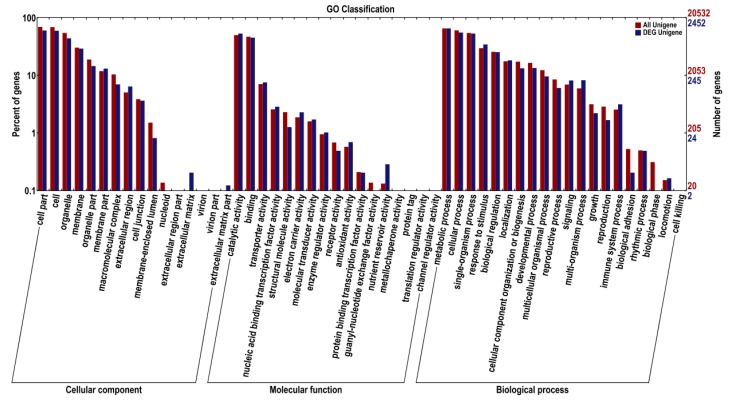
Functional gene ontology (GO) term classifications of DEGs from comparisons of WT and MT plants.

The global functional analysis of the DEGs was performed using GO annotation with Blast2GO, in order to derive ‘cellular components’, ‘molecular functions’, and ‘biological processes’. In the WT and MT comparisons, ‘cell parts’, ‘cell’, and ‘organelles’ were the top three categories involved in the ‘cellular components’; in the ‘molecular function’ category, ‘catalytic activity’, ‘binding’, and ‘transporter activity’ revealed the biggest differences; in the ‘biological processes’ category, the ‘metabolic processes’, ‘cellular processes’, and ‘single-organism processes’ showed the largest differences. (Figure 3).

#### 2.1.3. COG Enrichment and KEGG Pathway Analysis of DEGs

The COG (Cluster of Orthologous Groups) of proteins database is based on bacteria and algae. The eukaryotic system evolutionary relationships built using the COG database can classify the orthologous gene products. The top four categories for DEGs are (1) general function prediction only; (2) signal transduction mechanisms; (3) transcription; and (4) replication, recombination, and repair (Figure S2). These can be used to indicate the differences in the transcription factors and/or metabolic activity.

**Figure 4:**
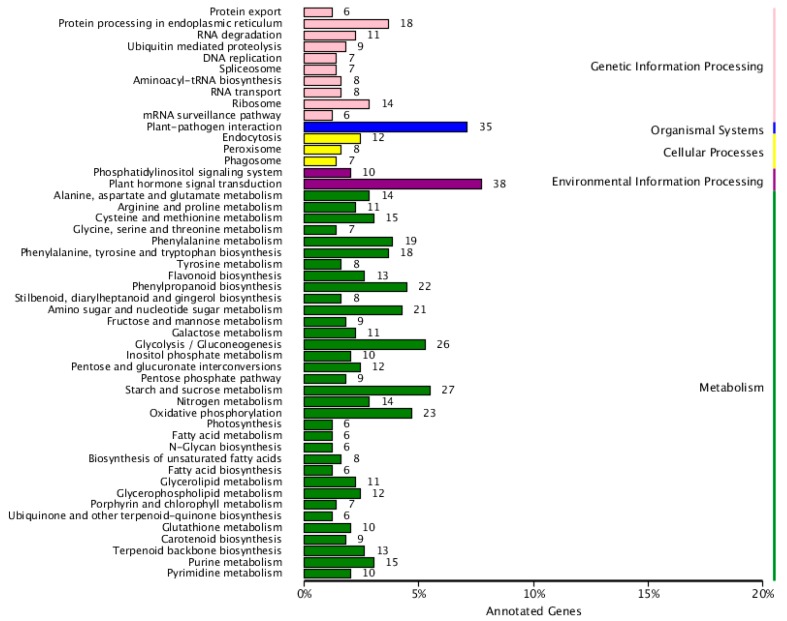
Kyoto Encyclopedia of Genes and Genomes (KEGG) classification of DEGs in the comparisons of WT and MT plants.

When in vivo, the gene products perform their biological functions through a series of interactions. The DEGs identified were next assessed using the KEGG (Kyoto Encyclopedia of Genes and Genomes) libraries to determine their higher-level functions and utilities in the biological system. From the identified DEGs, the database included 38 terms for ‘plant hormone signal transduction’ and 35 terms for ‘plant-pathogen interactions’ (Figure 4), which indicate that plant hormones may represent a key difference between the WT and MT plants. Furthermore, the ‘phenylpropanoid, tyrosine, and tryptophan biosynthesis’, ‘flavinoid biosynthesis’, and ‘nitrogen metabolism’ pathways displayed a high KEGG enrichment (Figure S3).

From our analysis, we have speculated that phytohormone may contribute to the differential phenotypes of the WT and MT plants. Coincidently, a 10-fold increase in the *CL13560contigle* (*SmGASA4*) expression was observed in the WT, compared to the MT plants (WT =1759 vs. MT = 173) (Table S6). Using NCBI, *SmGASA4* was found to possess a GASA conserved domain. *GASA* is an important GA regulated gene that contributes to root and flower development. This was the only DEG related to phytohormones that was identified from our analysis.

#### 2.1.4. Validation of DEGs by qRT-PCR Analysis

To validate our expression profiling, 18 DEGs were randomly selected and their expression was assessed by qRT-PCR (real-time quantitative PCR). Our results indicated identical patterns of expression for all of the 18 DEGs validating the transcriptome datasets (Figures S4 and S5).

### 2.2. Investigating the Role of SmGASA4 in Arobidopsis and S. miltiorrhiza

#### 2.2.1. Transgenes Expression in *Arobidopsis* and *S. miltiorrhiza*

**Figure 5:**
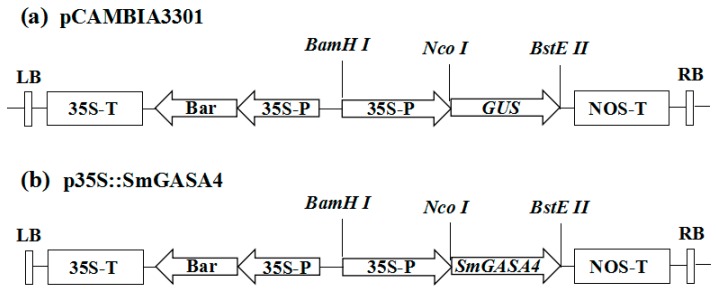
T-DNA regions of the binary plasmids used for Agrobacterium tumefaciens-mediated transformation, as follows: (**a**) Construct of the binary vector pCAMBIA3301, and (**b**) p35S::SmGASA4 binary plasmid containing Bar and SmGASA4, driven by the 35S promoter, 35S-P; the cauliflower mosaic virus-CaMV35S RNA promoter, 35S-T; CaMV35S poly A; NOS-T, the 3′ terminator region of the nopaline synthase; Bar; phosphinothricin (R). RB—right border; LB, left border.

To examine the role of *SmGASA4*, plasmids for its exogenous expression in *Arobidopsis* and *S. miltiorrhiza* were produced. Under the control of the constitutive CaMV35S promoter, p35S::SmGASA4 (Figure 5) were constructed. A total of four independent transgenic lines of *Arobidopsis* were obtained through the Basta-resistant screening and molecular identification (the *SmGASA4* primers are shown in Table S7). Three trans-gene lines, termed SmGASA4-1, SmGASA4-3, and SmGASA4-4, showed high levels of expression (Figure S6a), and the T4 homozygous overexpression plants were produced through a three generation purify. Four independent Basta-resistance lines of *S. militiorrhiza* were identified (the *SmGASA4* combination of primers are shown in Tables S7 and S8) and used to produce three further lines, termed SmGASA4-1, SmGASA4-3, SmGASA4-4. All were confirmed to be positive for the transgene (Figure S6b).

#### 2.2.2. *SmGASA4 Arobidopsis* and *S. miltiorrhiza* Phenotypic Observations

**Figure 6:**
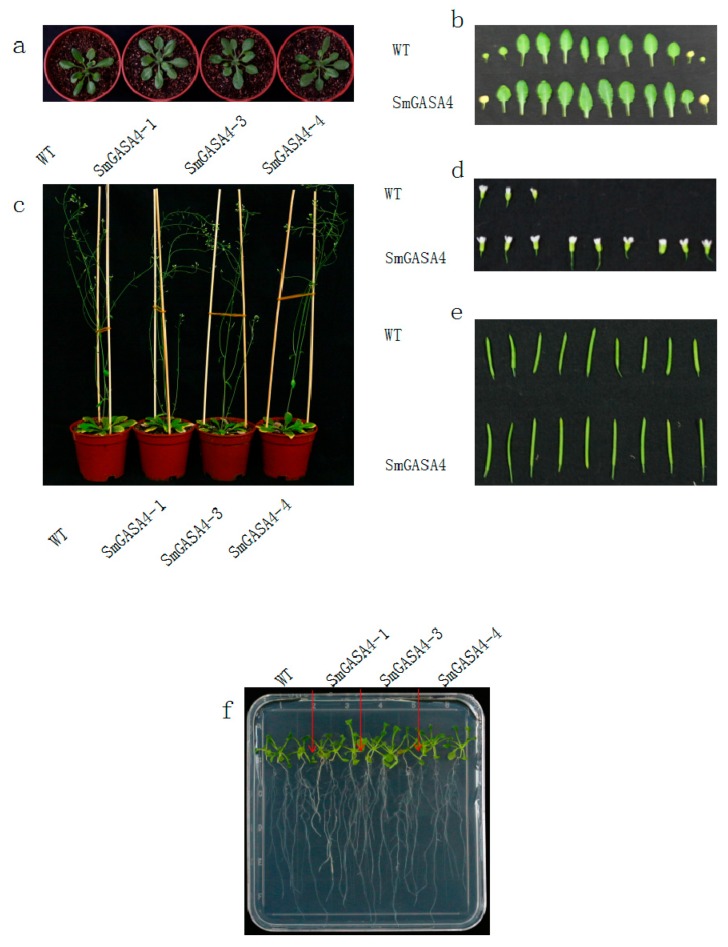
*SmGASA4 Arobidopsis* phenotypic observations, as follows: (**a**) 12 leaflet old *Arobidopsis*, (**b**) WT and *SmGASA4* leaves, (**c**) ripe period old *Arobidopsis*, (**d**) WT and SmGASA4 flowers, (**e**) WT and SmGASA4 capsules, (**f**) WT and SmGASA4 roots.

**Figure 7:**
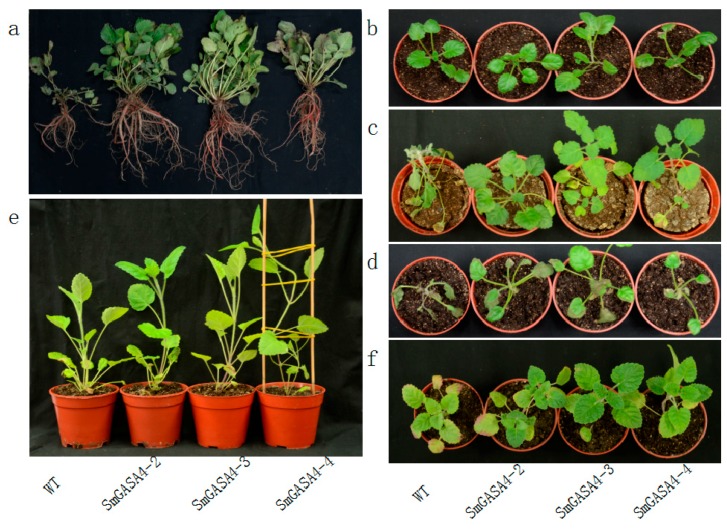
Effects of *SmGASA4* overexpression during *S. militiorrhiza* drought stress, as follows: (**a**) 90 day old *S. miltiorrhiza* phenotype, (**b**) *S. miltiorrhiza* before stress, (**c**) 30 day *S. miltiorrhiza* drought stress, (**d**) 4 day *S. miltiorrhiza* 250 mmol/L NaCl stress, (**e**) 29 day *S. miltiorrhiza* 200 μmol/L GA3 treatment, and (**f**) 29 day *S. miltiorrhiza* 300 μmol/L PBZ stress.

*Arobidopsis* typically grow 12–14 leaflets before bolting (Figure 6a). These leaflets were larger in the *SmGASA4*-*Arobidopsis* plants compared to the WT plants (Figure 6b). This suggested that *SmGASA4* promotes *Arobidopsis* growth during the seedling stage. No significant differences in either the plant height or leaflet size were observed between the WT and *SmGASA4* plants when the *Arobidopsis* had matured (Figure 6c). When the blossom periods of the WT and *SmGASA4 Arobidopsis* flowers were compared (Figure 6d), the *SmGASA4* flowers were larger (*SmGASA4 Arobidopsis* mature capsules were 1.25 times larger than the WT) (Figure 6e). In the MS (Murashige and Skoog) culture medium, the *SmGASA4-Arobidopsis* possessed larger lateral root densities than the WT (Figure 6f). The overexpression of *SmGASA4* in *S. miltiorrhiza* led to larger taproots (fold increase over WT: 1.92), and the lateral root number and length were also larger in *SmGASA4-S. miltiorrhiza* (fold increase over WT: 1.76 and 2.13, respectively) (Figure 7a).

#### 2.2.3. Overexpression of *SmGASA4* and Its Effects on Plant Drought Stress

**Figure 8:**
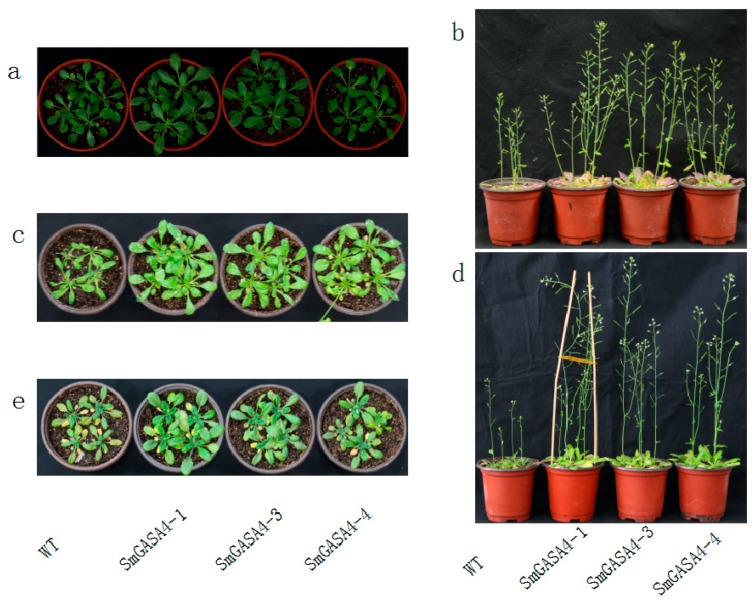
Effects of *SmGASA4* overexpression during *Arobidopsis* stress treatment, as follows: (**a**) *Arobidoposis* before stress, (**b**) 30 day drought stress, (**c**) 10 day 200 mmol/L NaCl stress, (**d**) 16 day 200 μmol/L GA3 treatment, and (**e**) 10 day 100 μmol/L paclobutrazol (PBZ) stress.

We next investigated whether the overexpression of *SmGASA4* can promote *Arobidopsis* tolerance to drought. WT and *SmGASA4-Arobidopsis* plants were subjected to identical periods of drought stress, and the plant growth was assessed (Figure 8a). During drought-stress, the *SmGASA4-Arobidopsis* growth was largely unaffected, whist the WT growth and development were severely inhibited. After 30 day of drought stress, the overexpression of the *SmGASA4* was found to complement the *Arobidopsis* life cycle, whilst the WT plants displayed a dwarf phenotype (Figure 8b). The overexpression of *SmGASA4* also led to a 1.54-fold increase in the plant height compared with WT, and an increased capsule number was observed (2.46-fold increase over WT). When the plants were grown in a 400 mmol/L mannitol MS solid culture medium, both the WT and *SmGASA4* growth and leaflet production were severely inhibited, but the root growths were prosperous (Figure S7a). The overexpression of *SmGASA4* still produced plants with improved growth conditions and lager lateral root densities than the WT plants (root length is 1.68 times that of the WT), which tended to wither in these conditions.

Across an identical timeframe, when the WT and *SmGASA4 S. miltiorrhiza* where compared (Figure 7b), the WT plants began to wilt after 28 day of drought stress, which accelerated to complete wilting on day 30. In contrast, no obvious changes as well as a lack of wilting were observed in the *SmGASA4* plants (Figure 7c). Following the water recovery, both the WT and *SmGASA4* plants displayed a normal recovery. These data suggest that the effects of SmGASA4 overexpression during drought stress are similar between *S. miltiorrhiza* and *Arobidopsis*.

#### 2.2.4. Overexpression of *SmGASA4* and Its Effects on Plant Salt Stress

To investigate whether *SmGASA4* can promote plant tolerance to salt, plants were sprayed with 200 mmol/L NaCl solution and the phenotypical differences in the growth characteristics of WT and *SmGASA4-Arobidopsis* were compared. After 10 day of salt stress, the WT plants displayed wilting, which was less apparent in the *SmGASA4* plants (Figure 8c). When 100 mmol/L NaCl was included in the culture medium, both the WT and *SmGASA4* plant leaflets narrowed and reduced lateral root lengths were observed (Figure S7b). These reductions were less apparent in the *SmGASA4* plants, which were larger than their WT counterparts. After 3 day spray with 250 mmol/L NaCl, the WT plants began wilting (Figure 7d), and both the WT and *SmGASA4 S. miltiorrhiza* leaves were severely damaged. These data suggest that, in both *S. miltiorrhiza* and *Arabidopsis*, the *SmGASA4* expression can improve the plants’ responses to salt stress.

#### 2.2.5. Overexpression of *SmGASA4* and Plant GA3 Responses

After 16 day of water and spraying with 200 μmol/L GA3 solution, the height of *SmGASA4-Arobidopsis* was 2.63-fold higher than the WT plants, and the capsule number increased by 9.17-fold, compared with the WT (Figure 8d). The overexpression of the *SmGASA4* gene also led to an increased stem length and early blooming times. It should be noted that the WT stem length and height were increased by GA3, and the effects were not as pronounced as those of the *SmGASA4* plants, consistent with the known effects of GA on *SmGASA4.* When 100 μmol/L GA3 was added to the culture medium, the *SmGASA4 Arobidopsis* plants displayed more leaflets and larger increases in lateral root density than the WT plants. The *SmGASA4* plants also displayed flowering and bolting at earlier timepoints (Figure S7c). After 29 day of 200 μmol/L GA3 spraying, the overexpression of *SmGASA4* in the *S. miltiorrhiza* plants led to increased plant heights and longer stems than the WT plants (Figure 7e). *SmGASA4 S. miltiorrhiza* displayed slight changes in leaf color, most likely attributed to their rapid growth characteristics. These results confirm constitutively that the expression *SmGASA4* can promote plants’ responses to GA3 treatment in both *S. miltiorrhiza* and *Arabidopsis*.

#### 2.2.6. Overexpression of *SmGASA4* and Its Effects on PBZ Induced Stress

PBZ is a potent inhibitor of plant growth due to its ability to inhibit GA biosynthesis. After 10 day of water and spraying with 100 μmol/L PBZ, the growth of the WT and *SmGASA4 Arobidopsis* plants was severely inhibited (Figure 8e). The *SmGASA4 Arobidopsis* plants displayed reduced stem lengths and deep color changes within the leaflets, with lesser purple of leaf bottoms, which began to transition to yellowness. The WT plants, by comparison, had a tendency to die following PBZ treatment. When 100 μmol/L PBZ was included in the culture medium, the *SmGASA4 Arobidopsis* plants survived longer than their WT counterparts, and whilst some plant death was evident, several *SmGASA4 Arobidopsis* plants maintained their green leaflets and remained alive (Figure S7d). After 30 day of 300 μmol/L PBZ spraying, both the WT and *SmGASA4 S. miltiorrhiza* stem lengths were reduced, but the leaflet formation was normal and bolder leaf veins were evident in the *SmGASA4* plants (these veins were more pronounced than the plants grown under normal conditions). The WT plants had more abundant purple/yellow transitions in the bottom leaflets, shorter stems, and a reduced plant height compared with the *SmGASA4* plants. Taken together, these data suggest that resistance to PBZ-induced plant death is conferred by *SmGASA4* overexpression (Figure 7f).

#### 2.2.7. Effects of *SmGASA4* Overexpression on Secondary Metabolism

We next assessed the effects of *SmGASA4* overexpression on the key genes involved in the tanshinone and phenolic acid biosynthetic pathways. qRT-PCR was performed in the WT and *SmGASA4* plants (Table S9), and the results were analyzed using iQ5 software (Figure S8). The qRT-PCR data revealed that the SmGASA4 expression led to a downregulation of *AACT*, *HMGS*, *MK*, *PMK*, *MDS*, *DXS*, *DXR*, *MCT*, and *MCS*; a modest upregulation of *HMGR* and *CMK*; and no significant differences to the *HDS* and *HDR* genes expression. Thus, it is apparent that *SmGASA4* inhibits the tanshinone biosynthetic pathway. For the genes involved in the phenolic acid biosynthetic pathway, *4CL1*, *C4H1*, *CPR1*, *HPPR1*, *PAL1*, *RTGus*, and *TATA1* were all significantly up-regulated by the expression of SmGASA4, suggesting that it promotes phenolic acid synthesis. Taken together, these data indicate that the *SmGASA4* expression can influence the secondary metabolism in *S. miltiorrhiza,* most notably enhancing the genes involved in phenolic acid biosynthesis.

## 3. Discussion

The medicinal benefits of *S. miltiorrhiza* mainly originate from the root surface. This has been well documented, with gene products that can alter metabolism, biosynthesis, and stress resistance reported [35,36]. Few studies have, however, focused on methods to improve root development, which would present obvious benefits to the biomass production of the medicinal components [7,8]. Here, we have performed transcriptome analyses in the MT plants that display one less lateral root as well as defects in flowering. After completing the sequencing of the genomes of the WT and MT, we obtained 11.69 GB of data (Table S1), 50,587 unigenes, WT 37,387 unigenes, and MT 34,416 unigenes (Figure S1, Table S2). Of these genes, 30,000 were taken for COG, GO, KEGG, KOG, Pfam, Swiss-Prot, and nr annotation (Table S4). The datasets revealed 5085 DEGs, 3714 of which were up regulated in the WT plants, and 1371 that were down regulated (Table S5). We annotated 3980 of these genes (Table S10). From the transcriptome analyses, volcano plots, MA, and hierarchical clustering analyses were performed to identify the key genes that led to the MT phenotype. From the KEGG analysis, we grouped the major changes into the ‘plant hormone signal transduction’ categories, and phytohormone was suspected to contribute to the mutant phenotype. Coincidently, a GA regulated DEG *SmGASA4* was significantly downregulated in the MT plants, with no other DEGs of the five phytohormones affected. As a plethora of studies have demonstrated that the GA and *GASA* genes play important roles in plant roots [37], stems [38], and flowering [14,16], we reasoned that *SmGASA4* may play a major role in the growth of *S. miltiorrhiza* and may contribute to the mutant phenotype.

The overexpression of *SmGASA4* was found to promote *Arobidopsis* development in the seedling period (Figure 6a,b). When overexpressed in mature *Arobidopsis,* no significant differences in leaf size or plant height were observed (Figure 6c). Whilst the overexpression of *AtGASA14* in *Arobidopsis* was found to promote plant rosette leaf development only during seedling [14], its overexpression in *S. militiorrhiza* promoted development for its entire lifecycle (Figure 7a). This effect may be due to genetic differences or the differential expression of *SmGASA4* regulatory genes. The *SmGASA4* expression was also shown to promote *Arobidopsis* flower and capsule development (Figure 6d,e), whilst its expression in *S. militiorrhiza* enhanced lateral root size and density compared to the WT plants (Figure 7a). Other genetic analyses revealed that the suppression of both *GASA4* and *GASA6* leads to late flowering, while the overexpression of *GASA6* leads to early flowering in *Arabidopsis,* consistent with our findings [39]. In addition, the *Arobidopsis* seeds from *GASA4–1*, null mutant plants display significantly reduced seed weight, whilst the seeds from the 35S::GASA4-overexpressing lines have significantly increased seed weights [24]. *AtGASA4* has been shown to regulate floral meristem identity and also positively affects both the seed size and total seed yield [16]. The previous characterization of *AtGASA4* showed that its promoter is active in the shoot apex, developing flowers, and developing embryos [16]. The *GASA* genes promote the growth of plant roots, rosette leaves, florals, capsule development, and seed products, which may be due to their high tissue and temporal specificity. An immunoblot analysis has also confirmed the presence of the *GASA4* gene product in flower buds, seedlings, and roots [32].

The meristem identity genes activate class A (*AP1* and *AP2*), class B (*AP3* and *PI*), and class C (*AG*) floral organ identity genes in pattern discrete regions of the flower. LEAFY (*LFY*), *AP1*, and *UFO* genes promote the *Arobidopsis* floral meristem identity, and the increased branching phenotype of plants with reduced *GASA4* expression are similar to those observed in *LFY* mutants [40]. *LFY* and *AP1* represent the key floral meristem identity genes, which specify lateral meristems to form flowers, but axillary inflorescent shoots [41,42]. *LFY* is a key integrator of signals that promote flowering, including the gibberellin pathway [43]. *AP2* regulates the flower meristem and organ identity, and development in *Arabidopsis.* What is more, *AP2* can also determine seed development, such as seed size, weight, and the accumulation of oil and protein in seeds, to a large extent [44]. The expression of *GASA4* and *GASA6* is dysregulated in plants with altered *LFY* [45] or AP3/PI activity. *GASA4* is involved in the regulation of hypocotyl elongation and flowering, in response to the integration of light signaling [46] and GA [47], which may suggest that these *GASA* genes regulate flower meristem and flower organ development.

*GASA5* is a negative regulator of GA-induced flowering and stem growth, and is known to delay flowering through enhancing the expression of the flowering locus C (*FLC*), and repressing the expression of the key flowering genes, including flowering locus T (*FT*) and *LFY* [38]. However, *GASA4*, *GASA6*, and *GASA14* are known to promote plant development [32,48]. The *GASA* gene expression displays species, tissue, and period specificity, and *GASA* may function through its regulation of transcription factors, including the GA receptor DIG1 [49], and GAI [48]. *GASA* is also regulated by other phytohormones, including Auxin [50] and ABA [12], amongst others. Further work is required to fully dissect the relationship between *GASA* and *LYF*, *AP1*, *AP2*, *AP3*, *PI*, *AG*, *UFO*, *FLC*, *FT*, DIG1, GAI.

Drought is one of the greatest environmental constraints to agriculture worldwide [51,52], The *AtDREB1* gene families can significantly promote *S. miltiorrhiza* drought tolerance [35,36]. *Snakin/GASA* proteins play an important role in plant resistance to abiotic stress, for example, the upregulation of *PagGASA* in poplar provides a high tolerance to drought stress [53]. In this study, the overexpression of *SmGASA4* promoted the *Arobidopsis* resistance to drought (Figure 8b and Figure S7a), an effect that was conserved in *S. miltiorrhiza* (Figure 7c). This represents the first example of the contribution of *GASA* genes to drought resistance in *S. miltiorrhiza*, demonstrating that the effects of *SmGASA4* on *Arobidopsis* are translatable to this system. *SmGASA4* contains a single CRP domain that can inhibit ROS accumulation, but the interactions that mediate its resistance to drought stress are still not fully characterized [32]. *GASA* contains 1–5 ABRE components, meaning that the enhancement of plant resistance to drought could be mediated by the ABA (abscisis acid) pathway or through the modulation of SA (salicylic acid) levels [54,55]. However, the relationship between Sm*GASA4* and Ca^2+^, *IP3*, *DAG*, *PA*, *SOS*, *CBF*/*DREB1*, *DREB2*, and *ARF*/*AREB* remains unclear [56,57].

In *Arobidopsis,* the overexpression of *AtGASA14* can promote salt stress resistance [14]. *OsGASR1* expression is induced by salt or ABA treatment, and the ectopic expression of *OsGASR1* enhances salt resistance via the inhibition of ROS [58]. In this study, *SmGASA4* overexpression also promoted the salt resistance of *Arobidopsis* (Figure 8c and Figure S7b), and reduced plant wilting in salt stress conditions in both *Arobidopsis* and *S. miltiorrhiza* (Figure 7d). The overexpression of *AtGASA14* was also found to suppress reactive oxygen species (ROS) accumulation, a function that is required for its GASA domain [32]. The overexpression of the GA-responsive gene, *FsGASA4*, also enhances salt, oxidative stress, and heat stress tolerance during seed germination by means of increasing the SA biosynthesis. GA may thus play a crucial role during early plant responses to adverse environmental conditions by modulating the SA levels [54,55]. It is currently unclear whether *SmGASA4* enhances salt resistance through a similar mechanism.

The *Snakin*/*GASA* genes are involved in plant growth and stress tolerance by regulating DELLA expression [38,59]. DELLA is a negative regulator of GA signaling [33] and components of the GA signaling pathway influence plant growth [60]. GA regulates the expression of *GASA1*, *GASA4*, *GASA6* and *GASA9* through DELLA expression [33], with *GASA2*, *GASA3*, and *GASA4* upregulated in response to exogenous GA3 [32,61]. In this study, *SmGASA4* had a positive response to the GA3 treatment (Figure 8d and Figure S7c) in *Arobidopsis*, as it promoted plant root, leaflet, stem, flower, and capsule development. Similar results occurred in *S. miltiorrhiza* following GA3 treatment, however slight leaf color changes were observed, most likely due to the enhancement of growth (Figure 7e). The *GASA* gene family contains a stretch of 12 conserved cysteines, and its promoter sequences include 1–4 GARE components, which facilitate its participation in the GA response. *GASA2*, *GASA3*, and *GASA4* are upregulated in response to GA3 [32,61]; *GASA5* is inhibited by GA3, but induced by ABA; *GASA9* is inhibited both by GA3 and ABA [33]; and the *SN1*, *GASA10*, *GASA12*, *GASA14,* and *GASA15* expression are unaffected by GA3 [12,17]. In *Arobidopsis, GASA4* and *GASA6* expression are most likely mediated through the GA unresponsive, GA INSENSITIVE (GAI) protein [48]. Gibberellin regulates the GASA-like gene, *GEG*; putative secretion; and the expression levels, which can also regulate cell wall division and extension [14,30]. GA, ABA, and Auxin may also interaction regulate *SmGASA4*; *OsGSR1* are downregulated by brassinosteroids (BR) [33,62]; and the interaction between *SmGASA4* and BR, GAI, and RGA should be further researched [32].

PBZ is an inhibitor of GA biosynthesis and thus is a potent inhibitor of plant growth. During *Arobidopsis* seed germination, *GASA14–1* plants showed an enhanced sensitivity to PBZ compared with the WT plants [14]. In this study, the overexpression of *SmGASA4* in *Arobidopsis* strongly promoted plant resistance to PBZ (Figure 8e and Figure S7d), whilst the WT plants displayed leaf discoloration and a tendency for plant death. The WT plants also displayed shorter stem lengths, reduced height, and more pronounced yellow and purple leaflets (Figure 7f). *S. miltiorrhiza* displayed a stronger resistance to PBZ in the culture medium than *Arobidopsis* seedlings (Figure S7d). PBZ severely inhibited *Arobidopsis* stem elongation, but did not influence the leaflet formation. In *Arobidopsis,* the expression of the *GASA4* gene, regulated by gibberellin, is tissue-specific. Indeed, it is up-regulated in the meristematic region, but inhibited in cotyledons and leaves [29]. Interesting, *GASA4* is up-regulated in meristematic region, but it is down-regulated in cotyledons and leaves by gibberellin. So, *GASA4* may be participate in cell division. It indicates that it is highly expressed in meristematic tissues with higher cell division rate, but is less expressed in differentiated tissues [29]. *GASA* genes display tissue specific expression and interactions with Auxin, ABA, and other phytohormone [48]. The ability of *SmGASA4* to promote plant resistance to PBZ may therefore be mediated through DELLA and ROS.

*GASA* has been shown to participate in secondary metabolism. In this study, we demonstrated that the *SmGASA4* expression in *S. miltiorrhiza* downregulates the key genes of the tanshinone biosynthetic pathway, whilst significantly upregulating phenolic acid biosynthetic genes (Figure S8). Tanshinone is a diterpenoid quinone compound that possesses a similar cyclizing theory in the initial geranylgeranyl diphosphate (GGPP) process with GA. Tanshinone biosynthesis may therefore compete with GA biosynthesis, meaning that the down-regulation of its biosynthetic genes by *SmGASA4* would increase the GA levels. This mechanism and the role of *SmGASA4* in *S. miltiorrhiza* secondary metabolism biosynthesis requires further investigation.

Taken together, our experiments partially reveal the phenotype of the MT mutation. GA may interact with Auxin, ABA, and BR [33,62], and so regulate *GASA* and plant development. It is unclear whether there is a potential interaction with TFs, such as *AtTZF1* [63], GAI [48], and RST-1 [50]. *GASA4* elevates heat tolerance [28,64]; *GsGASA1* can response to chronic cold stress [59]; *snakin-1* activity is induced by temperature changes and wounding [65]; *gid1* is involved in the tolerance to cold stress and resistance to blast fungus [66]; and *GASA5* negatively responses to heat stress through an inhibition of SA signaling, and reduces the antioxidant capacity and accumulation of heat shock proteins in *Arobidopsis* [38,67]. In *S. miltiorrhiza*, many *GASA* gene family members are unexpressed and their in vivo functions now require further investigation.

## 4. Materials and Methods

### 4.1. RNA-Seq and Library Construction

The total RNA was isolated from the plant roots using RNA extraction Kits (Eastep Super, Promega, Shanghai, China) and treated with RNase Free DNase I (New England BioLabs, Beverly, MA, USA). RNA quality/purity was assessed using the Nanodrop 1000 spectrophotometer (Thermo Fisher Scientific, Wilmington, DE, USA), and the RNA concentration and integrity assessed using a Qubit 2.0 Fluorimeter (Life Technologies, Carlsbad, CA, USA), and Agilent Bioanalyzer 2100 system (Agilent Technologies, Santa Clara, CA, USA). The mRNA was purified with Oligo (dT) magnetic beads. The fragmentation was randomly performed in an IIIlumina proprietary fragmentation Buffer. Using the mRNA as a template, random oligonucleotides synthesized the first-strand of cDNA, and the addition of dNTPs, RNase H, and DNA polymerase I synthesized the second-strand of cDNA, which was purified with AMPure XP beads. The purified double-stranded cDNA were enzymatically repaired, polyadenylated, and ligated to adapters. The fragment size selection was performed with AMPure XP beads and the cDNA libraries constructed through PCR amplification.

Qubit 2.0 tests were used to assess the library concentration and the Agilent 2100 test was used to assess insert sizes. Q-PCR was used to assay the concentration of the libraries, to ensure quality. The cDNA library was sequenced using Illumina HiSeq^TM^2500 (Vancouver, BC, Canada) with PE100.

### 4.2. De Novo Transcriptome Assembly and Functional Annotation

The raw data was filtered by removing the adapter sequence and low quality reads to produce high quality clean data. The sequence assemble of the clean data was assessed to produce Unigene libraries, most of the basic group quality score more than Q30, using the Trinity [16] software to assemble the clean data into Unigene.

### 4.3. Unigene Functional Annotation

BLAST software (available online: http://genome.ucsc.edu/cgi-bin/hgBlat) was used to compare the Unigene sequences with the non-redundant (nr) protein databases (nr, available online: ftp://ftp.ncbi.nih.gov/blast/db/), Swiss-Prot (available online: http://www.uniprot.org/), gene ontology (GO, available online: http://www.geneontology.org/), Clusters of Orthologous Groups (COGs, available online: http://www.ncbi.nlm.nih.gov/COG), euKaryotic Orthologous Groups (KOG, available online: http://www.ncbi.nlm.nih.gov/COG/), and Kyoto Encyclopedia of Genes and Genomes (KEGG, available online: http://www.genome.ad.jp/kegg/) libraries. Following prediction, the Unigene amino acid sequences were assessed using HMMER software (available online: http://hmmer.janelia.org/) for assignment into protein families (Pfam, available online: http://pfam.xfam.org/), to obtain Unigene annotation information.

### 4.4. Quantification of Gene Expression

The clean data were mapped to the unigene library using Bowtie [68]. The read count for each gene was then obtained from the mapping results using RSEM [31]. The Fragments Per Kilobase of transcript per Million mapped read (FPKM) [37] values or each unigene were calculated to determine their expression profiles. The differential expression analysis of the WT and MT were analyzed using DESeq [38], with Benjamini and Hochberg False Discovery Rate (FDR) [69] methods. The DEGs were identified with a threshold of FDR <0.01 and fold change (FC) ≥2 or ≤−2. A cluster analysis was performed according to the patterns of unigene differential expression across the samples [70].

### 4.5. Sequencing Data Verification and Secondary Metabolism Analysis

The total RNA was extracted from the *S. miltiorrhiza* roots and treated with RNase Free DNase I (TaKaRa, Dalian, China). The reverse transcription was performed using SuperScript III (RT kit; Invitrogen, Carlsbad, CA, USA), following the manufacturer’s recommendations. A total of 18 DEG genes were randomly chosen to verify the RNAseq data (Table S11), using primer 5 to design the 18 DEG specific primers (Table S12). A quantitative reverse-transcription PCR (qRT-PCR) analysis was performed on an iQ5.0 instrument (Bio-Rad, Hercules, CA, USA) with SYBR Green qPCR kits (Roche, Basel, Switzerland) by heating at 95 °C for 3 min, followed by 40 cycles at 95 °C for 30 s, and 60 °C for 30 s. The amplification reactions (20 μL) consisted of 10 μL 2× SYBR Green Mix, 1 μL cDNA, and 0.25 μM forward and reverse primers. The relative gene expression levels were calculated using the 2^−ΔΔ*C*T^ method and normalized against Actin (Table S8). All assays for a particular gene were performed in triplicate under identical conditions. Using primer 5 design, the 28 *S. miltiorrhiza* secondary metabolism biosynthesis genes (Table S9) were assessed using identical qRT-PCR methods.

### 4.6. Plasmid Construction

The full-length *SmGASA4* open reading frame was amplified using PCR (polymerase chain reaction) from Transcriptome cDNA, with primers containing the restriction sites *NcoI*/*BstEII* (Table S7). Both of the PCR products were cloned into the pEASY-T1 vector (TransGen Biotech, Beijing, China). Digestion was performed, and the *SmGASA4* fragment was inserted into the binary plant vector pCAMBIA3301 containing the CaMV35S constitutive promoter (Figure 5). The Bar gene encodes glufosinate acetyltransferase (PAT), which is resistant to the herbicide glufosinate (PPT), so it is used to be considered as a screening marker (Figure 5). The constructed vector was transformed into Agrobacterium tumefaciens, strain LBA4404, by heat-shock methods.

### 4.7. Plant Materials and Genetic Transformation

*Arabidopsis* (Ecotype Columbia) transformation was carried out using the floral dip method [71]. We obtained transgenic line T4, which can stablely inherit using glufosinate resistance screening and molecular identification methods.

The leaves of the WT and MT plants were cultured on MS (Murashige and Skoog) + 1.0 mg/L 6-BA + 1.0 mg/L NAA (Naphthaleneacetic acid) culture medium, containing 30 g/L sucrose and 6 g/L agar after surface sterilization with 0.1% HgCl_2_ [64]. After the Callus differentiation breed is translated into a MS + 1.0 mg/L 6-BA + 0.01 mg/L NAA culture medium, last translate into ½ MS + 0.2 mg/L IBA culture medium to induce root formation. The cultures were placed in a growth chamber under specific conditions, with temperature between 23 and 27 °C under a 16 h light/8 h dark photoperiod (light intensity = 150 μmol m^−2^ s^−1^). Each vector was transfered into *Salvia miltiorrhiza* by the leaf-disk method, mediated by Agrobacterium tumefaciens [64]. The *A. tumefaciens*, strain LBA4404, stored in 50% glycerol water, was transferred to a liquid yeast extract (YEB) medium for activation at 28 °C and cultured on a shaker (200 rpm) to mid-log phase (OD600 = 0.6). The excised leaves were pre-incubated on MS agar for 2 day at 25 ± 2 °C and 16-h-light/8-h-dark photoperiod.

The pre-cultured leaves were placed in the bacterial-containing liquid for 25 min, and the excess liquid was blotted on a sterile filter paper, and then placed the leaves on MS agar with 1.0 mg/L 6-BA + 0.01 mg/L NAA in the dark. The temperature was maintained at 25 ± 2 °C. After 2 day of co-cultivation, the leaves were placed on selective medium with 0.6 mg/L Basta and were cultured under specific conditions (25 ± 2 °C, 16 h light/8 h dark, and 150 μmol m^−2^ s^−1^). The leaf disks were sub-cultured every week. The shoots were generated after 35 day and subsequently were excised and transferred to selective rooting medium. Two weeks later, the rooted plantlets were cut into sections and then cultured on ½ MS basal medium for propagation. A half-month-old seedling can be used for PCR screening and gene expression evaluation. The transformed plants were grown in the soil for further observations and stress treatment.

### 4.8. Molecular Characterization of Transgenic Events

The CTAB method was used to extract the genomic DNA from the young leaves [67] of *Arobidopsis*. Positive p35S::SmGASA4 transgenic lines were detected based on the sequences of the *SmGASA4* promoter (Table S7). *S. miltiorrhiza* RNAs were extracted from young leaves using the Eastep Super (Promega, Shanghai, China), following the manufacturer’s instructions, and were translated into cDNA. The primer sets for the p35S::SmGASA4 transgenic lines were verified using the 35S promoter sequence of pCAMBIA3301 (Table S8) for the forward primer, and SmGASA4-R in *SmGASA4* sequence for the reverse primer (Table S7).

### 4.9. Stress Treatment

To analyze the *Arobidopsis* response to drought, NaCl, GA, and PBZ stress, as well as six-leaved plantlets and bolting date seedlings were individually transferred to sterile soil containing nutrients, after the seedling had adapted to the new environment. Prior to drought stress, both WT and transgene plants were saturated with water and left overnight to drain. Salt stress was performed using 200 mmol/L NaCl solution and sprayed onto the plant. GA stress was performed with 200 μmol/L GA3 solution, and PBZ stress with a 100 μmol/L PBZ solution sprayed onto the plants. Sterile *Arobidopsis* seeds were added to ½ MS medium, after the seedling four-leaved transform to the stress ½ MS culture medium. The drought culture medium contained 400 mmol/L mannitol, salt culture medium contained 100 mmol/L NaCl, GA culture medium contained 100 μmol/L GA3, and PBZ culture medium contained 50 μmol/L PBZ. All of the treatments were performed over a 16:8 h photoperiod (light intensity = 150 μmol m^−2^ s^−1^), 22 ± 2 °C.

To analyze the *S. miltiorrhiza* response to drought, NaCl, GA, and PBZ stress, as well as plantlets in solid sterile medium were transferred to sterile nutrient-containing soil, and then placed in a growth chamber for cultivation (25 ± 2 °C, 16 h light/8 h dark, light intensity = 150 μmol m^−2^ s^−1^). Once the plantlets had adapted to their new environment, the stress treatments were initiated. Prior to drought stress, both the WT and transgene plantlets were saturated with water and left overnight to drain. The pants were sprayed with 250 mmol/L NaCl (salt stress), 200 μmol/L GA3 (GA stress), or 300 μmol/L PBZ (PBZ stress). GA3 and PBZ were dissolved with ethyl alcohol prior to being dissolved in water.

## 5. Conclusions

Our laboratory produced a mutant plant that possessed a single huge taproot, reduced lateral roots, and defective flowering. By performing a transcriptome analysis, we found that phytohormone signaling may play an important role during WT plant growth. We identified a significant DEG, *SmGASA4*, which displayed a reduced expression in the MT plants. The overexpression of *SmGASA4* was subsequently shown to promote root development and promote flower and capsule development in *Arobidopsis*. We further demonstrate that *SmGASA4* is a *GASA* family member that is positively regulated by GA. *SmGASA4* expression was also shown to improve plant resistance to drought, salt stress, and PBZ. Finally, we demonstrate that the overexpression of *SmGASA4* in *S. miltiorrhiza* can upregulate the key genes of the salvianolic acid biosynthesis, but down regulates the genes involved in tanshinone biosynthesis.

## Supplementary Materials

Supplementary materials can be found at http://www.mdpi.com/1422-0067/19/7/2088/s1.

## Abbreviations

*S. miltiorrhiza*	*Salvia miltiorrhiza*
WT	Wild type *Arobidopsis*/*Salvia miltiorrhiza*
MT	Mutant type *Salvia miltiorrhiza*
*GASA*	Gibberellic acid-stimulated *Arabidopsis*
GA	Gibberellin
PBZ	Paclobutrazol
PPT	Herbicide phosphinothricin
PAT	Phosphinothricin acetyltransferase
GARE	Gibberellin Acid Response Element
ABRE	Abscisic Acid Response Element
DEGs	Differentially expressed genes
qRT-PCR	Real-time quantitative PCR
MS	Murashige and Skoog
SA	Salicylic acid
