# Supplementary material for: Transcriptome Analyses from Mutant Salvia miltiorrhiza Reveals Important Roles for SmGASA4 during Plant Development
Source: Int J Mol Sci. 2018 Jul 18;19(7):2088. doi: 10.3390/ijms19072088 (PMC6073587; doi:10.3390/ijms19072088)
Supplement: Supplementary file 1 [file ijms-19-02088-s001.pdf]

International Journal of *Molecular Sciences*

MDPI

Article

Transcriptome Analyses from Mutant *Salvia miltiorrhiza* reveals important roles for *SmGASA4* during plant development

Hongbin Wang [1], Tao Wei [1], Xia Wang[1], Lipeng Zhang[1], Meiling Yang[1], Li Chen[1], Wenqin Song[1], Chunguo Wang[1,*], Chengbin Chen[1,*]

[1] College of Life Sciences, Nankai University, Tianjin 300071, China; 2120150901@mail.nankai.edu.cn (H.W.); 8190537@nankai.edu.cn (T.W); 2120160946@mai.nakai.edu.cn(X.W); 1120140344@mail.nankai.edu.cn (L.Z.); meilingyang@tju.edu.cn (M.Y); lichen@nankai.edu.cn (L.C); songwq@nankai.edu.cn (W.S.)

* Correspondence: wangcg@nankai.edu.cn (C.W.); chencb@nankai.edu.cn (C.C.); Tel.: +86-22-2350-8800 (C.C.)

Received: date; Accepted: date; Published: date

Supplementary Materials:

1. Supplementary Figures

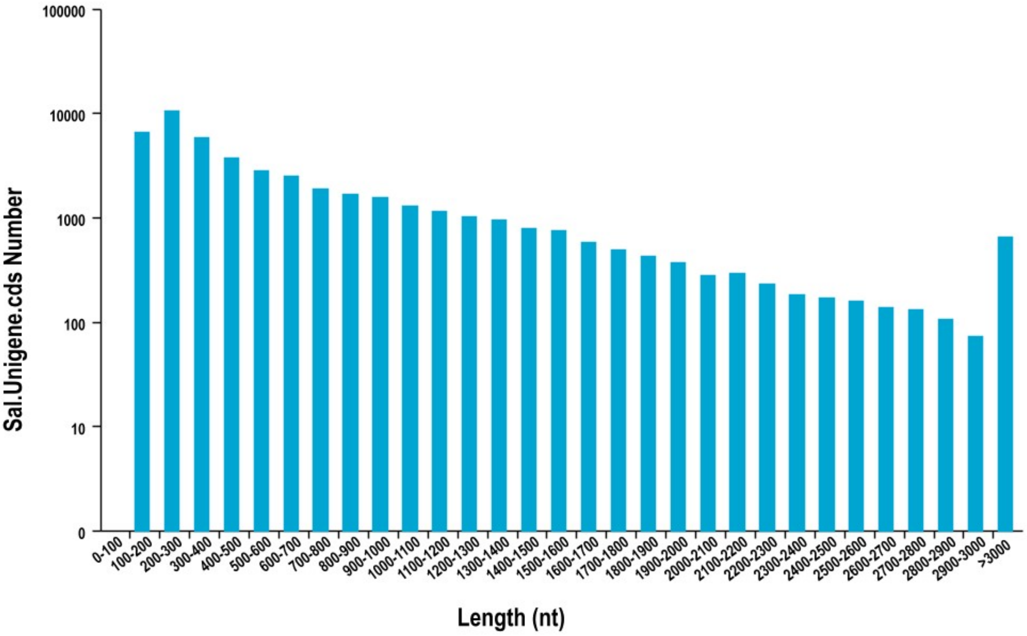

Figure S1. Sequence length distribution of Unigenes in WT and MT plants.

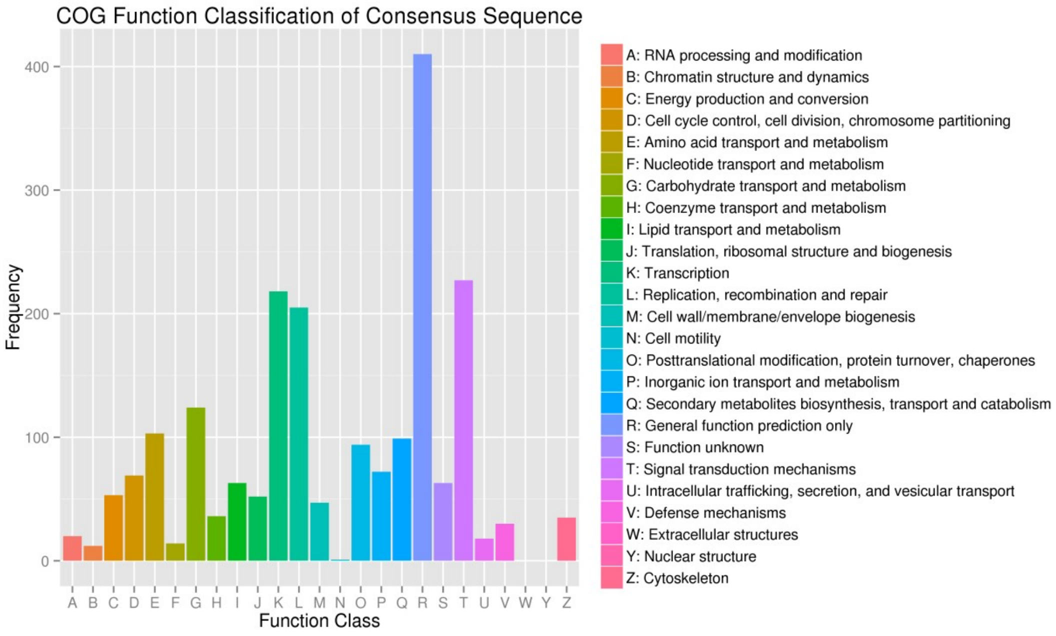

Figure S2. COG functional Classification of DEGs in the WT and MT plants.

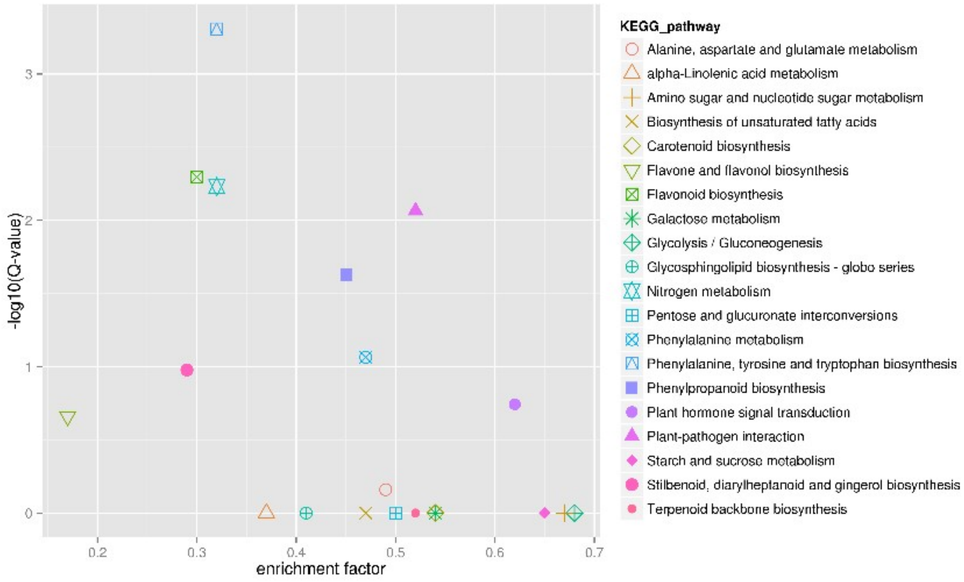

Figure S3. Scatter plot of KEGG pathways enriched in the comparisons of WT and MT plants.

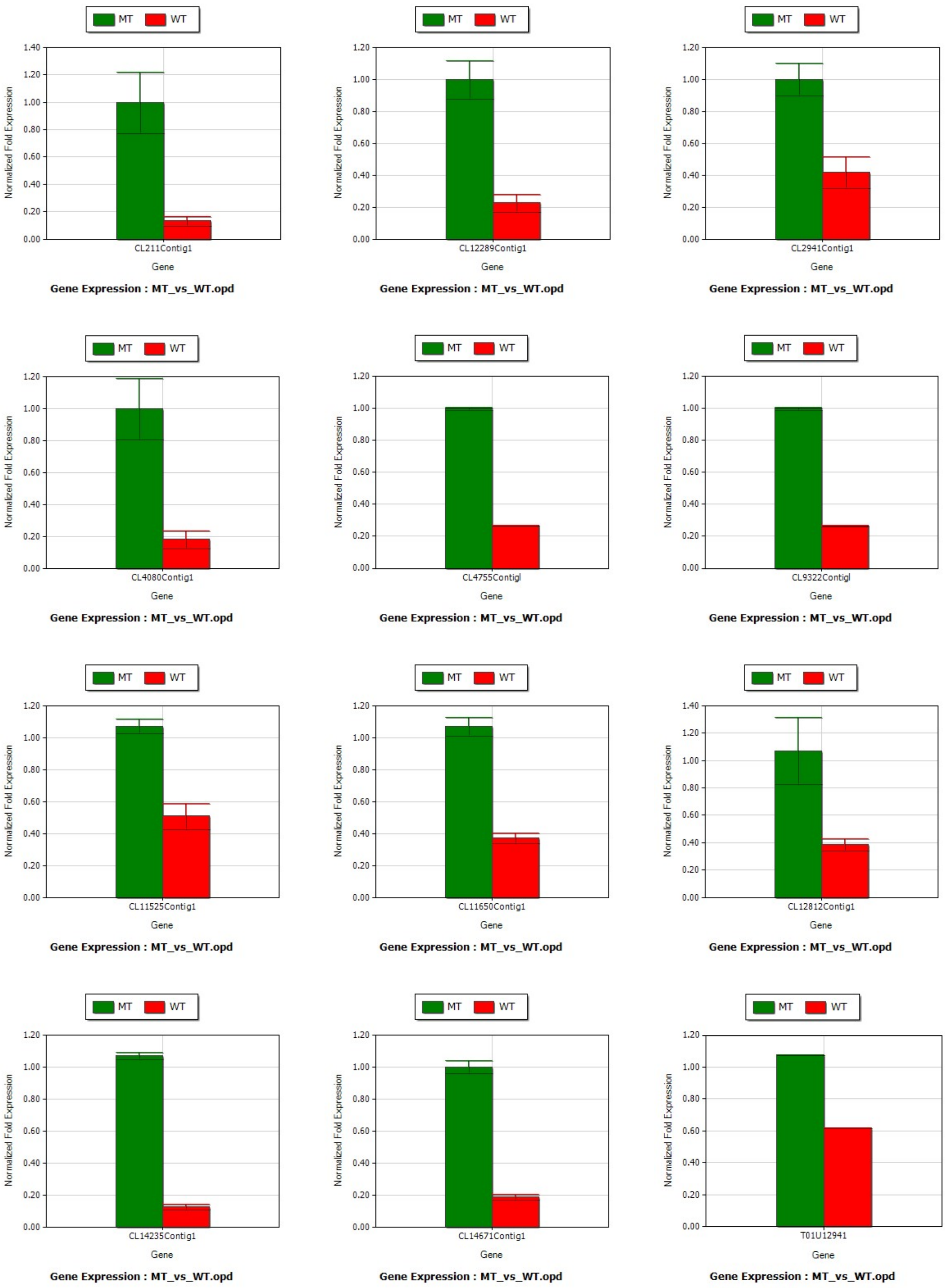
MT WT
Normalized Fold Expression
CL211Contig1
Gene
Gene Expression : MT_vs_WT.opd
CL12289Contig1
CL2941Contig1
CL4080Contig1
CL4755Contigl
CL9322Contigl
CL11525Contig1
CL11650Contig1
CL12812Contig1
CL14235Contig1
CL14671Contig1
T01U12941

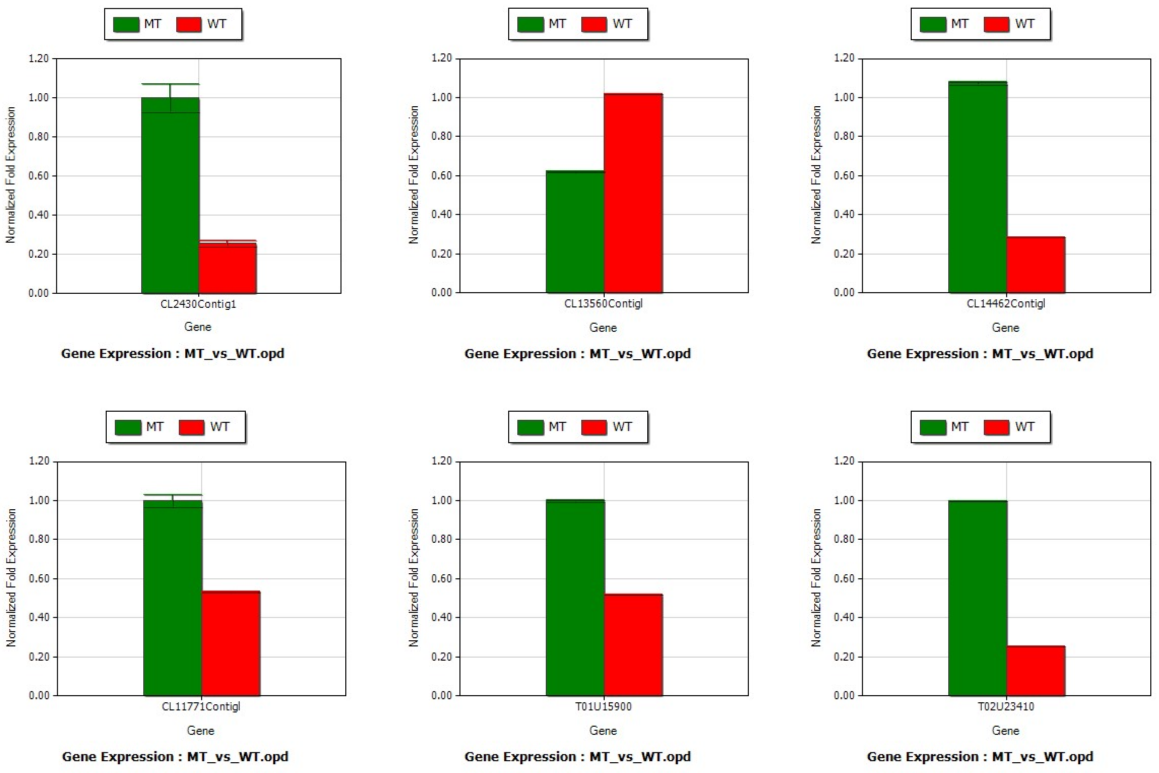

Figure S4. Validation of 18 DEGs by qRT-PCR Analysis.

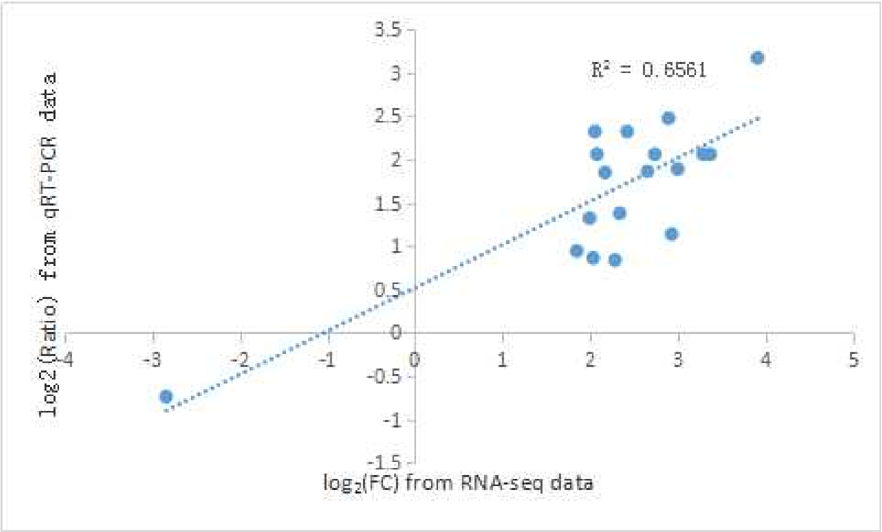

Figure S5. Correlations in changes in gene expression between fold-change determined from RNA-seq data (X-axis) and data obtained using qRT-PCR (Y-axis).

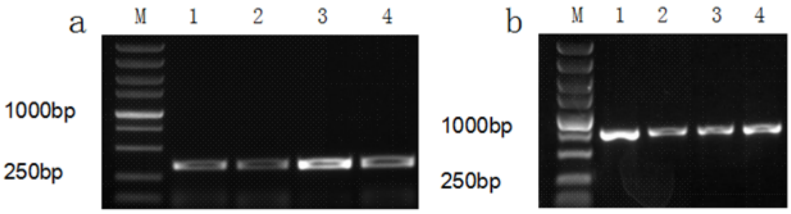

Figure S6. Identification of transgenic SmGASA4 *Arabidopsis* and *S.miltiorrhiza* plants via PCR: (a) *Arabidopsis*: PCR analysis of primary transformants using specific primers for SmGASA4-F::SmGASA4-R. Lanes 1, size markers; lanes 2-5, DNA from putative transformants *Arabidopsis* (b) *S.miltiorrhiza*: PCR analysis of primary transformants using specific primers for p35S::SmGASA4. Lanes 1, size markers; lanes 2-5, cDNA from putative transformants *S.miltiorrhiza.* M: TAKALA 5000 Marker.

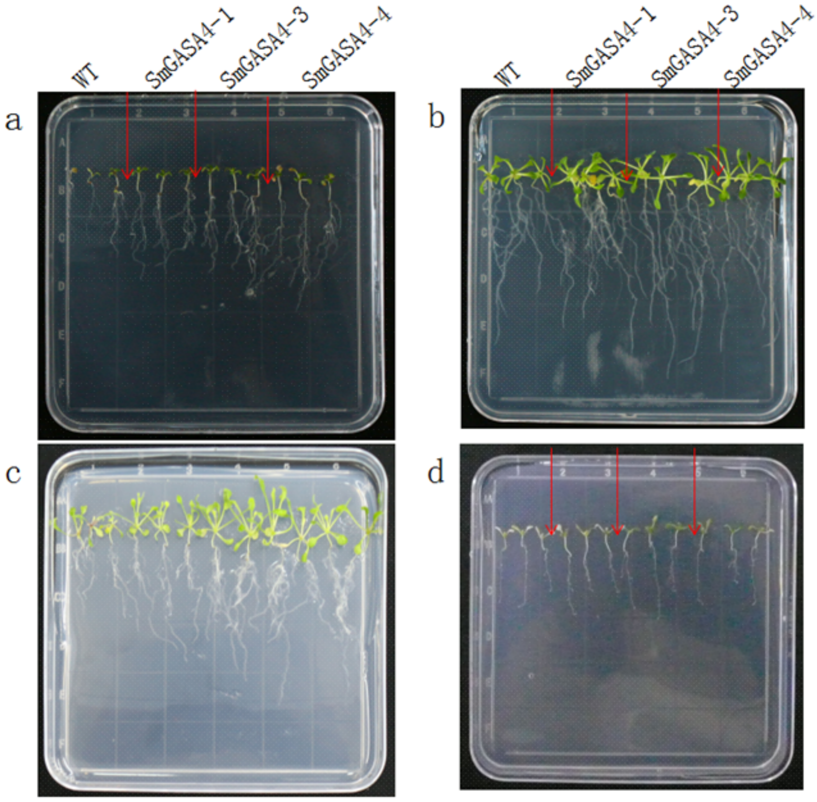

Figure S7. Effects of *SmGASA4* overexpression during *Arabidopsis* stress treatment: (a) 400 mmol/L mannitol drought stress; (b) 100 mmol/L NaCl stress; (c) 100 μmol/L GA3 treatment; (d) 100 μmol/L PBZ stress.

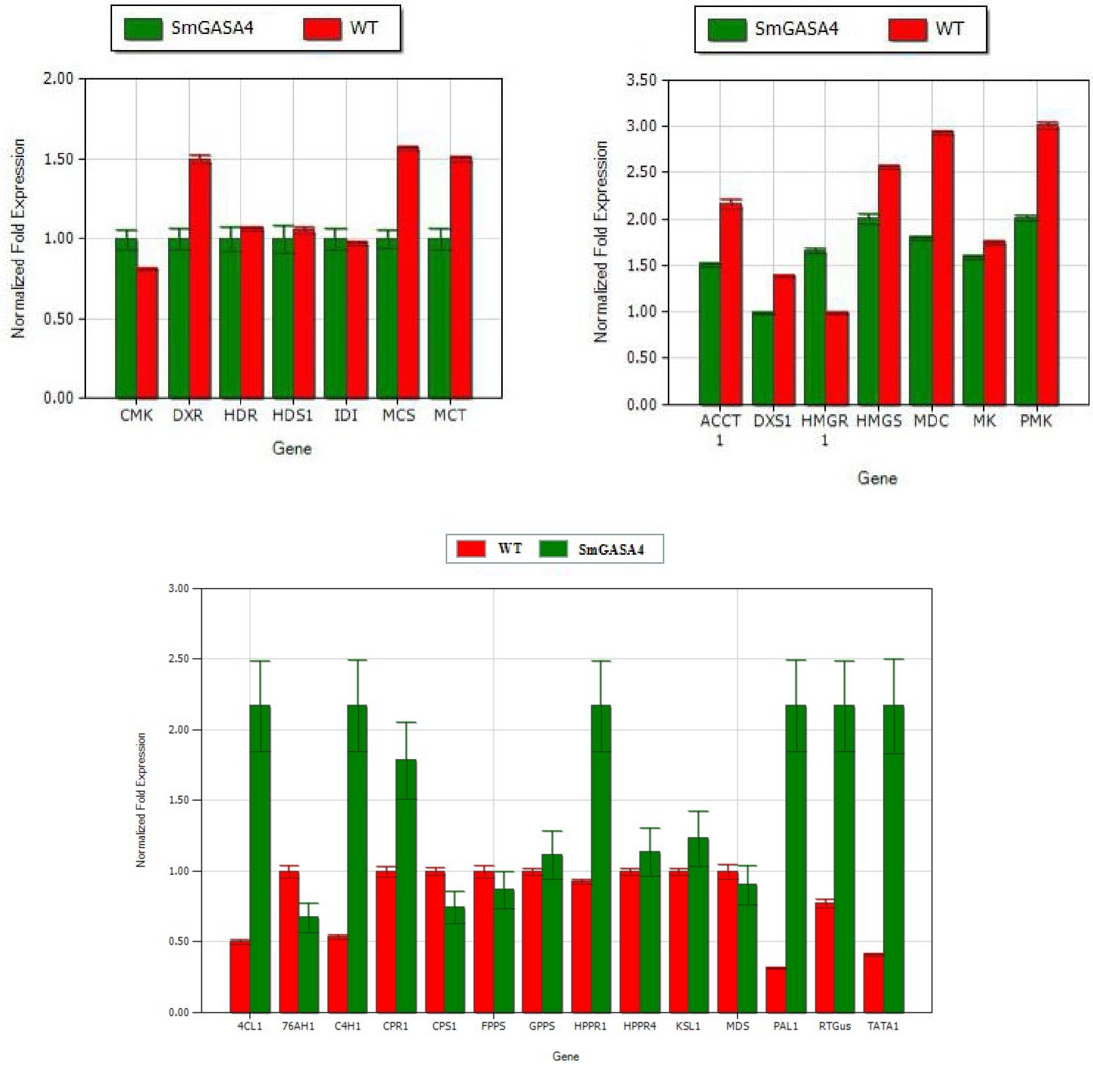

Figure S8. Effects of *SmGASA4* overexpression on secondary metabolism by qRT-PCR Analysis.

2. Supplementary Tables

Table S1. Sequencing data statistics for the WT and MT *S.miltiorrhiza* RNA-seq libraries.

Samples	BMK-ID	Read Number	Bsae Number	GC Content	%≥Q30
M-RI	T01	27,255,174	5,504,953,429	50.18%	91.58%
WT-R3	T02	30,598,611	6,180,267,382	49.74%	91.83%

Table S2. Assembly results of the WT and MT *S.miltiorrhiza* transcriptome.

Length Range	T01 Unigenes	T02 Unigenes	All Unigennesis
200-300	9,109(26.47%)	10,756(28.77%)	13,749(27.18%)
300-500	7,364(21.40%)	8,281(22.15%)	10,563(20.88%)
500-1000	7,127(20.71%)	6,979(18.67%)	9,584(18.95%)
1000-2000	6990(20.31%)	7,025(18.79%)	10,065(19.90%)
2000+	3,826(11.12%)	4,346(11.62%)	6,626(13.10%)
Total Number	34,416	37,387	50,587
Total Length	31,275,399	33,699,085	48,417,894
N50 Length	1,540	1,610	1,698
Mean Length	908.75	901.36	957.12

Table S3. Summary of the transcriptome assembly.

BMK-ID	Clean Reads	Mapped Reads	Mapped Ratio
T01 (MT)	27,255,174	21,538,490	79.02%
T02 (WT)	30,598,611	24,135,294	78.87%

Table S4. Annotation results of the assembled unigenes.

Annotated databases	Unigene	≥300nt	≥1000nt
COG	10,115	9,606	7,066
GO	20,532	18,326	11,575
KEGG	6,337	5,839	4,023
KOG	17,343	15,590	10,271
Pfam	21,674	20,211	14,014
Swiss-Prot	21,304	19,170	12,120
nr	29,805	26,481	16,090
All	30,000	26,605	16,113
Annotated databases	Unigene	≥300nt	≥1000nt

Table S5. Statistics of DEGs in the WT and MT.

DEG Set	All DEG	up-regulated	down-regulated
WT_vs_MT	5,085	3,714	1,371

Table S6. Annotation the DEGs of *CL13560contigl* (*SmGASA4*).

#ID	T02 (WT)	T01 (MT)	FDR	log2FC	regulated	Annotation
CL13560Contig1	1758.794207	172.9985793	1.28E-05	-2.841616313	down	Gibberellin regulated protein

Table S7. Sequence-specific primers used for the *SmGASA4* Plasmid construction.

SmGASA4-F	CCATGG	NcoI	GGCCATGGATGGCTTATCTTTATCGTC
SmGASA4-R	GGTNACC	BstEII	GGTGACCTTAAGGGCACTTGGTCTT

Table S8. Other oligo nucleotide primers used in this study.

Oligo name	Primer-F (5'-3')	Primer-R (5'-3')
Actin	GGTGCCCTGAGGTCCTGTT	AGGAACCACCGATCCAGACA
35S	AACAGAACTCGCCGTAAAG	TAGTGGGATTGTGCGTCAT

Table S9. Key genes of tanshinone and salvianolic acid biosyntheses sequence-specific primers used for qRT-PCR.

Oligo name	Primer-F (5'-3')	Primer-R (5'-3')
CMK	ACCGTGGCTCCTCGTCTTTAC	CGGAATCCCAGCATCCCTAT
DXR	CGCTGCGTTTGCCTATTCTGT	CGCTTTCTCATTGGCTGCACTA
HDR1	GCATTGGCGGATGGAACTC	CCCTCTTTCTCCACCAACTCG
HDS	GGAGCCTTTCAGACCGCATTA	ACCCAAGTGTCCCGATTCCTA
IDI	GGTTGTCGTCCTTGACCAGC	GCGTTGAGCGGAGAAATCG
MCS	GGAGGAGGCAGTTCGGCTAAT	AGAGGTTCACAACGGAAGGGTC
MCT	GGGTGTTGGGTGTTCCTGCTA	GGGCGTCATCGGTAACTTCG
AACT1	CGCAGAGGCGAGGAAAGGA	CACGCAAAGGCACCGACAT
DXS1	CCCGTGTTCAATGCTCCTCA	CGAACTCGGCATCCTGTCTC
HMGR1	TGAGGCTGCAAGGCAATCTATG	GCTTCAACTCTGTCGCCCTCTT
HMGS	ACTTTGGCTGGGCAGAGGGT	TGTGGGAACTCGTGCCTTGAC
MDC	CCGCCGATAGTAATCAGTTCCA	CCGCATCAAAGGTATAAGCCAC

MK	CGGGGTAGACAATACAGTAAGCAC	CCGAGCATCTTCAAAGGCATA
PMK	GGGTTCTGCTGGCTGGTGT	CCGAGGGTCGTTGCTTTCTA
4CL1	GGCTGCCGTTGTGCCTATG	TCGCCACCTGGTTCGCTAT
CYP76AH1	CACCCAACTTCGCCGACTACTT	CCCGCCTGAATAATATCAACCAC
C4H1	CCGCCCAGGAGTCCAAAT	CGAGCCACCAAGCGTTCA
CPR1	GAGCCGCAGGATGAGAAGACC	CGTCTCCATATGAGGCAAGGAAG
CPS1	TGGATGGGCAGCAGCAGTA	GCGGCGACACGCTTATTC
FPPS	GCCCTGCTGGTTTAGAGTCCC	CCAATGCGGCGATGAAGAG
GPPS	CCCTTTCGCAATCCTATTCAGTC	GGGAACCTCCGCAACTACCAT
HPPR1	CCAGCGTGGGATTGGACAG	CCCTCAGAACCGCCAGCAT
HPPR4	TCCTACGAATCTCCGCTCCC	CCACGGCAATCAACAACCC
KSL1	GGGTGATTACTTGTCTGCCTCC	GGGTTTTCTTTGCGTTCCTTC
MDS	GGAGGAGGCAGTTCGGCTAAT	AGAGGTTCACAACGGAAGGGTC
PAL1	CGCCAGCAGTGATTGGGTTAT	TGGTTGGATTCTGTCCCGTTTC
RTGus	GCTGCTGTCGGCTTTAACCTC	GCTCTTTAATCGCCTGTAAGTGC
TAT1	GTAATGCGGCTCAGGAAGGTG	GGGCGGACAATGCTATCTCAAT

Table S10. Annotation results of the assembled DEGs.

DEG set	Annotated	COG	GO	KEGG	KOG	Pfam	Swiss-Prot	nr
MT_vs_WT	3,980	1,297	2,452	681	2,041	3,023	2,956	3,945
DEG set	Annotated	COG	GO	KEGG	KOG	Pfam	Swiss-Prot	nr

Table S11. 18 DEGs for WT and MT sequencing data verification.

S.miltiorrhiza unigene ID	Log2FC	Regulated	Description
CL211contigl	2.89	up	protein-arginine deiminase activity
CL12289contigl	3.37	up	HAD superfamily, subfamily IIIB (Acid phosphatase)
CL2941contigl	1.99	up	galactinol-raffinose galactosyltransferase activity
CL4080Contigl	2.42	up	iron ion binding，electron carrier activity
CL4755Contigl	2.99	up	Posttranslational modification, protein turnover, chaperones
CL9322Contigl	3.29	up	Nematode resistance protein-like HSPRO2
CL11525Contigl	2.93	up	4-coumarate-CoA ligase activity
CL11650Contigl	2.65	up	regulation of transcription, DNA-templated
CL12812Contigl	2.33	up	plant-type cell wall organization,cell wall modification
CL14235Contigl	3.91	up	Late embryogenesis abundant protein
CL14671Contigl	2.05	up	response to salt stress ,detection of ethylene stimulus
T01U12941	2.28	up	Proline-rich receptor-like protein kinase PERK9
CL2430Contigl	2.73	up	unsaturated fatty acid biosynthetic process,response to cold
CL13560Contigl	-2.84	down	gibberellin-regulated protein 14-like [Solanum lycopersicum]
CL14462Contigl	2.17	up	hydroxypyruvate reductase activity, glyoxylate reductase (NADP) activity
CL11771Contigl	2.03	up	chorismate biosynthetic process,response to herbicide
T01U15900	1.85	up	transcription, DNA-templated
T02U23410	2.07	up	Secondary metabolites biosynthesis, transport and catabolism

Table S12. 18 DEGs sequence-specific primers used for WT and MT sequencing data verification.

***S.miltiorrhiza* unigene ID**	**Primer-F (5'-3')**	**Primer-R (5'-3')**
CL211contigl	GGTGATAAGAAGCGGGACG	GGCTGGTTGCTGTTGGGT
CL12289contigl	CTATCCCTTCCGCCACCC	CCACGCTTTCCAACCTTCCT
CL2941contigl	CGTCGTCAACATCGCAAAGG	CCGCAGCAGCCAAGTAGC
CL4080Contigl	TCAAGCCGCTCCAGTTCC	GCCATAGTCCGCCCAAAT
CL4755Contigl	CGATGATAGCGAACCACTCCT	GCGATTGTTTACTGGCTTCTTGT
CL9322Contigl	GCACCTTCTCCAGCAACCAG	GGACGCTCTACTCCACGCATC
CL11525Contigl	GCGAAGCAGCCACTACCAA	GCCTCCGCATCATTCAAGTA
CL11650Contigl	GCCTCCAGTTCCAGCAAAGC	CGCCAGGATAGTCCGTCAGTT
CL12812Contigl	CGGTCTGCGGGCTGTTGA	GCCACGCCGACTTCTCCA
CL14235Contigl	CGCCAGCGATTCTCCACC	CGTTTCCGAGCAGCACCAA
CL14671Contigl	CCTGTGCGAGAATCTGGGACT	CCGCCGATGTTGATGACGA
T01U12941	CGGGCTATCTCCGCCAGTA	GCGGCAGGTTTGTCTTCG
CL2430Contigl	CGTGGAATGGCAGAGGGC	GCGGGCTTACGACACTTGA
CL13560Contigl	CACCGCCTTTCACCACCA	CAGGGACGAGTAGTCATCCAGAC
CL14462Contigl	GGCGTGGGATTGGACAGA	CCTCAGAACCGCCAGCAT
CL11771Contigl	CGTACAAGTCTCCTGGGAAAGC	CCTGGCGACATCTGGCATT
T01U15900	CAAATCCATTCCAATCCTCGTT	GGTGGCGGCATTATCATCG
T02U23410	CATGGCTACGGCGACTCTG	GGATAATCCTAAGCGGAACTGC

© 2018 by the authors. Submitted for possible open access publication under the terms and conditions of the Creative Commons Attribution (CC BY) license (http://creativecommons.org/licenses/by/4.0/).
